# Chromium-Salophen as a Soluble or Silica-Supported Co-Catalyst for the Fixation of CO_2_ Onto Styrene Oxide at Low Temperatures

**DOI:** 10.3389/fchem.2021.765108

**Published:** 2021-10-29

**Authors:** Matthieu Balas, Ludivine K/Bidi, Franck Launay, Richard Villanneau

**Affiliations:** ^1^ CNRS UMR 7197, Laboratoire de Réactivité de Surface, LRS, Campus Pierre et Marie Curie, Sorbonne Université, Paris, France; ^2^ CNRS UMR 8232, Institut Parisien de Chimie Moléculaire, IPCM, Campus Pierre et Marie Curie, Sorbonne Université, Paris, France

**Keywords:** heterogeneous catalysis, CO_2_ utilization, cycloaddition reaction, styrene oxide, chromium salophen complexes

## Abstract

Addition of a soluble or a supported Cr^III^-salophen complex as a co-catalyst greatly enhances the catalytic activity of Bu_4_NBr for the formation of styrene carbonate from styrene epoxide and CO_2_. Their combination with a very low co-catalyst:Bu_4_NBr:styrene oxide molar ratio = 1:2:112 (corresponding to 0.9 mol% of Cr^III^ co-catalyst) led to an almost complete conversion of styrene oxide after 7 h at 80°C under an initial pressure of CO_2_ of 11 bar and to a selectivity in styrene carbonate of 100%. The covalent heterogenization of the complex was achieved through the formation of an amide bond with a functionalized {NH_2_}-SBA-15 silica support. In both conditions, the use of these Cr^III^ catalysts allowed excellent conversion of styrene already at 50°C (69 and 47% after 24 h, respectively, in homogeneous and heterogeneous conditions). Comparison with our previous work using other metal cations from the transition metals particularly highlights the preponderant effect of the nature of the metal cation as a co-catalyst in this reaction, that may be linked to its calculated binding energy to the epoxides. Both co-catalysts were successfully reused four times without any appreciable loss of performance.

## Introduction

Global warming remains one of mankind’s greatest challenges and, today, it is imperative to find environmentally friendly and cost-effective solutions to limit the increasing concentration of greenhouse gases in the atmosphere. (IPCC. Climate Change, 2014) For this reason, chemistry offers innovative solutions that encourage us to consider CO_2_ not as a waste but also as a source of high valued compounds. (Aresta et al., 2013). However, its conversion is not an easy task and requires catalysts, thus allowing its utilization as a C_1_ precursor. Among the various targets considered in the literature, the formation of cyclic carbonates with a rather biodegradability through the reaction of CO_2_ with epoxides generates actually a growing interest. Indeed, cyclic carbonates have a wide range of applications such as monomers in plastics, solvents in paints, batteries, and even degreasers, or as organic intermediates for the synthesis of dimethylcarbonate (Kamphuis et al., 2019). Furthermore the so-called cyclocarbonatation reaction of epoxides represents a green alternative to one of the conventional synthesis of carbonates based on the phosgenation of diols used industrially since 1833 (Fukuoka et al., 2003).

It is noteworthy that this particular reaction requires the use of organocatalysts (Guo et al., 2021), generally quaternary ammonium salts, but the addition of metal complexes or salts as well as hydrogen bond donors acting as Lewis acids (co-catalysts) (Kim et al., 2013; Adhikari et al., 2014; Supasitmongkol and Styring, 2014; Wang et al., 2014; Sharma et al., 2018; Rehman et al., 2021; Zhou et al., 2021), allowing the activation of epoxides, is a strategy often implemented (Darensbourg and Holtcamp, 1996; Laugel et al., 2013; He et al. 2014; Comerford et al., 2015; Claver et al., 2020). Among the various co-catalysts used, transition metal complexes with Schiff-base ligands (salen or salophen) represent a particularly attractive class of compounds due to their ease of synthesis and the large variety of metal centres that can be incorporated within their N_2_O_2_ coordination sphere (Yoon and Jacobsen, 2003; Cozzi, 2004; Gupta and Sutar, 2008; Decortes et al., 2010). Their structure allows the introduction of a large range of substituents that can influence the Lewis acidity of the metal centre. Furthermore, it is also possible to use this chemical flexibility for the covalent immobilization of such catalysts onto a support. Excellent reviews have already been published on the subject (Baleizao and Garcia, 2006; Wezenberg and Kleij, 2008; Gupta et al, 2009).

While there are many examples of salen/salophen CO_2_ cycloaddition catalysts incorporating transition metal ions such as Mn^3+^ or Zn^2+^, those with Cr^3+^ are far fewer of them (Paddock and Nguyen, 2001; Alvaro et al., 2004; North et al., 2015; Castro-Osma et al., 2016).

Indeed, most studies in the literature reported their activity as efficient catalysts for the copolymerisation of oxiranes and CO_2_ to provide the corresponding polycarbonates (Darensbourg et al., 2006; Darensbourg et al., 2008; Li et al., 2007; Niu et al., 2009; Veronese et al., 2020), a process that was predicted to be more kinetically favorable than the formation of the corresponding cyclic carbonate in the case of chromium salen complexes (Darensbourg et al., 2003).

However, the selective formation of cyclic carbonates in the presence of chromium salophen complexes was first described by Paddock and Nguyen in 2001 (Paddock and Nguyen, 2001). Since, chromium-salophen co-catalysts combined with various Lewis bases such as DMAP (Adhikari et al., 2014), Bu_4_NBr (North et al., 2015) or ionic liquids (Alvaro et al., 2004) have allowed the cycloaddition of CO_2_ under increasingly mild conditions, some working at room temperature or atmospheric pressure. As such, North and co-workers (North et al., 2015) reported a 89% yield of the carbonate of the 1,2-epoxy-3-phenoxypropane at room temperature and under 1 bar of CO_2_ within 24 h using a homogeneous dual catalysts system based on Bu_4_NCl and Cr^III^ Jacobsen-type salen complex in solvent-free conditions. However, in this case, the authors used 2.5% of Cr^III^ co-catalyst, which is a particularly large amount for a metal catalyst, especially in the case of a metal such as chromium.

In order to have an easy recovery by filtration, supported versions of those complexes are highly desirable. In this respect, different supports allowing the heterogenization of salen/salophen complexes were studied in the literature, among these are zeolites or mesoporous silica (Srivastava et al., 2003; Ayala et al., 2004; Kureshy et al., 2006; Silva et al., 2006; Kleij, 2009; Melendez et al, 2011). However even though immobilisation of chromium salen/salophen was already achieved with coordinative and covalent bonding for several catalytic applications (Canali and Sherrington, 1999; Gigante et al., 2000; Baleizão et al., 2002; Wang et al., 2007), only a few studies mention their use for the cycloaddition of CO_2_ onto epoxides, highlighting in this particular case the relevance of covalent anchoring (including the use of MOF) for better recyclability (Ramin et al., 2005; Zalomaeva et al., 2013; Xie et al., 2014). The present work focuses on the co-catalytic performances in the CO_2_ cycloaddition reaction onto styrene oxide of a Cr^III^ salophen complex bearing a carboxylic acid group, either in homogeneous conditions or after its covalent grafting onto propylamine-functionalized mesoporous silica of the SBA-15 type.

## Materials and Methods

The synthesis and the characterization of the ligand and of the Salophen-tBu-Cr complex can be obtained in the Supplementary Material, as well as the protocols for the catalysis tests in homogeneous and heterogeneous conditions and all the details on the different characterization techniques for the complexes and materials are reported.

The SBA-15 functionalized by 2.3 mmol of {NH_2_}.g^−1^ (TGA, c.a. 77% incorporation yield) ({NH_2_}-SBA-15) was obtained by the reaction of SBA-15 with (3-aminopropyl) triethoxysilane (APTES). After anchoring the chromium (III) complex, the material recovered was characterized by XRD, TGA (metal concentration of 0.8 mmol g^−1^ from Supplementary Figure S6) and N_2_ physisorption. Overall, a significant decrease of the specific surface area was observed after grafting as expected (see Supplementary Table S1 and Supplementary Figure S7) (from 900 m^2^ g^−1^ for SBA-15 to 530 for {NH_2_}-SBA-15, then 310 m^2^ g^−1^ for {Salophen-tBu-Cr}-SBA-15). This was accompanied by a concomitant decrease in pores volume (from 0.69 for {NH_2_}-SBA-15 to 0.44 cm^3^ g^−1^ for {Salophen-tBu-Cr}-SBA-15) and mean pores size values (from 5.7 nm for {NH_2_}-SBA-15 to 5.2 for {Salophen-tBu-Cr}-SBA-15). These data are compatible with those observed in the case of manganese and nickel complexes grafted on silica SBA-15 described elsewhere (Balas et al., 2021). Furthermore, {Salophen-tBu-Cr}-SBA-15 was characterized by a high hexagonal structuration of its porosity (presence of the (100), (110), and (200) reticular planes characteristic of the SBA-15 structure, see Supplementary Figure S8). It is noteworthy that the successive grafting of the propylamine groups, then of the salophen complex onto SBA-15 did not significantly alter the support.

## Results and Discussions

### Synthesis and Characterization of the Co-Catalysts

In the continuity of our previous work (Balas et al., 2021), the preparation, the characterization, and the immobilization of a Cr^3+^-salophen complex bearing a –CO_2_H function (N,N’-bis(3,5-di-tert-butylsalicylidene)-1-carboxy-3,4-phenylene-diamine-chromium (III)chloride) onto a mesoporous SBA-15 silica through amide bonding was thus addressed in the present manuscript. This compound showed excellent co-catalytic performances in the cycloaddition reaction of CO_2_ onto styrene oxide, both in its soluble form or after heterogenization.

**SCHEME 1:**
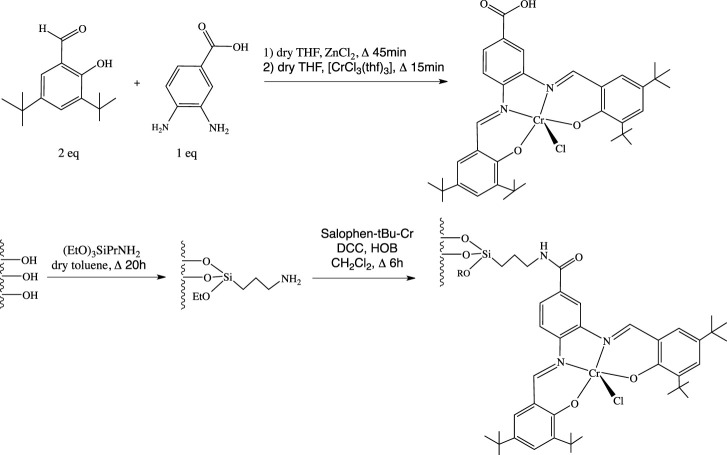
Synthesis steps of the co-catalyst and of its covalent grafting onto the surface of an amino functionalized silica support.

The ligand (Salophen-tBu, Scheme 1), i.e., N,N’-bis(3,5-di-tert-butylsalicylidene)-1-carboxy-3,4-phenylene-diamine was synthesized according to the experimental protocol developed by Hey-Hawkins and coworkers (Schley et al., 2010; see also Supplementary Figures S1, S3 for more details as well as operating protocols in the supplementary information section). Then, the chromium derivative, (Salophen-tBu-Cr), i.e., N,N’-bis(3,5-di-tert-butylsalicylidene)-1-carboxy-3,4-phenylene-diamine-chromium(III)chloride was easily prepared from the reaction of Salophen-tBu in distilled tetrahydrofuran (thf for short) with commercial [CrCl_3_(thf)_3_] under argon, leading to a 94% yield (see Supplementary Figures S4, S5 for the full characterization of the compound).

The presence of a –COOH function on the central phenyl group of the salophen ligand allowed to covalently graft the complex at the surface of an amino functionalized SBA-15 silica ({NH_2_}-SBA-15), according to our previous works on the anchoring of hybrid polyoxometalates (Villanneau et al.,2013; Bentaleb et al., 2015) and as shown also for the Mn and Ni derivatives (Balas et al., 2021). This occurred through the formation of a covalent amide bond using N,N’-dicyclohexylcarbodiimide (DCC) and 1-hydroxy-1H-benzotriazole (HOBT) coupling agents, in dichloromethane under refluxing conditions, affording Salophen-tBu-Cr@{NH_2_}-SBA-15. According to the comparison of the TGA profiles of {NH_2_}-SBA-15 and Salophen-tBu-Cr@{NH_2_}-SBA-15, the Cr loading could be estimated to 0.8 wt%.

### Catalytic Performances of Salophen-tBu-Cr and Salophen-tBu-Cr@{NH_2_}-SBA-15 in the Cycloaddition Reaction of Styrene Oxide With CO_2_

**SCHEME 2:**
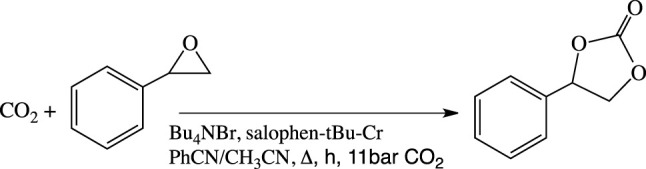
Cycloaddition reaction of CO_2_ onto styrene oxide.

The performances of the soluble and heterogenized forms of the chromium complex to act as co-catalysts were compared in the formation of styrene carbonate from styrene oxide and CO_2_ using tetra-butyl ammonium bromide as a catalyst (Scheme 2). The experiments were performed in an autoclave under 11 bar of CO_2_ (initial pressure) with temperatures as low as 50–80°C. The reaction products were quantified by Gas Chromatography and ^1^H NMR spectroscopy after 3, 7, and 23 h. In all experiments, styrene carbonate was found as the only reaction product and no polymeric materials could be detected. Consequently, the yield of styrene carbonate was equal to the styrene oxide conversion in this work.

The process was initially implemented using soluble tetrabutylammonium bromide (Bu_4_NBr) and Salophen-tBu-Cr, respectively, as the main catalyst and the Lewis acid co-catalyst, either in benzonitrile or acetonitrile and in the presence p-xylene (internal standard). Regarding our previous results and the corresponding literature, Bu_4_NBr was shown to have better catalytic performances for this particular reaction compared to other tetraalkylammonium salts bearing shorter chain lengths. Thus, at the highest reaction temperature tested (80°C), Bu_4_NBr alone (using a styrene oxide:Bu_4_NBr ratio = 112:2, corresponding to 1.8 mol%) led to a 12% yield of styrene carbonate after 3 h, the reaction being complete after 23 h (Table 1, entry 1). At the same time, Salophen-tBu-Cr (using a styrene oxide:co-catalyst ratio = 112:1, corresponding to 0.9 mol%) showed no activity for this specific reaction in the absence of NBu_4_Br at 80°C (Table 1, entry 2).

**TABLE 1:** Yield (%) of styrene carbonate obtained with Cr-salophen co-catalyst.

Entry	Co-cat (lewis acid)	cat. (Lewis base)	T (°C)	3 h	7 h	23 h
1	—	Bu_4_NBr	80	12	20	100
2	Salophen-tBu-Cr	—	80	n.d.	n.d.	0
3	Salophen-tBu-Cr	Bu_4_NBr	80	75	92	100
4	—	Bu_4_NBr	60	2	5	24
5	Salophen-tBu-Cr	Bu_4_NBr	60	26	47	96
6	—	Bu_4_NBr	50	0	2	11
7	Salophen-tBu-Cr	Bu_4_NBr	50	12	29	69
8	Salophen-tBu-Mn	Bu_4_NBr	80	n.d.	48	n.d.
9	Salophen-tBu-Ni	Bu_4_NBr	80	n.d.	26	n.d.
10^a^	Salophen-tBu-Cr	Bu_4_NBr	80	n.d.	91	n.d.

Conditions: Styrene oxide (5.6 mmol), Bu_4_NBr (0.1 mmol), soluble Salophen-tBu-M (0.05 mmol) in 13 ml of benzonitrile under 11 bars of CO_2_ (initial pressure).

a Reaction conducted in acetonitrile. n.d. = this reaction was not carried out under these specific conditions.

However, using Bu_4_NBr and Salophen-tBu-Cr together at once (styrene oxide:co-catalyst:Bu_4_NBr ratio = 112:1:2) led to a dramatic increase of the styrene oxide conversion at the early stages of the reaction with a 75% yield of styrene carbonate within 3 h at 80°C with 100% selectivity (Table 1, entry 3). At this temperature the reaction was almost complete (92%) after 7 h, while a conversion of 20% was observed with Bu_4_NBr alone.

Furthermore, the presence of the co-catalyst also allowed to obtain an important added value at lower temperature. An increase of the styrene oxide conversion at 60°C was thus observed after 23 h from 24% in the absence of Salophen-tBu-Cr to 96% in its presence (Table 1, entries 4 and 5). Finally, the styrene oxide conversion reached 69% after 23 h at 50°C (Table 1, entries 6 and 7) with both Salophen-tBu-Cr and Bu_4_NBr (11% in the absence of Salophen-tBu-Cr).

From this study, it appeared that the chromium (III) salophen complex behaved clearly as a better homogeneous co-catalyst compared to the corresponding manganese (III) and nickel (II) complexes (Balas et al., 2021). Indeed a higher yield of styrene carbonate was observed at 80°C for Salophen-tBu-Cr (92% after 7 h) compared to Salophen-tBu-Mn and Salophen-tBu-Ni (respectively, 48% for Mn and 26% for Ni) during the same reaction time (Table 1, entries 8 and 9). It is noteworthy that no styrene oxide conversion was observed with these two last co-catalysts at 50°C. This should be seen in conjunction with the DFT calculations performed by Deng and Lu’s team, which concern the interactions between different metal-salophen complex monomers and propylene oxide (Wu et al., 2017). Of all the metals tested, Cr(III) was indeed found to have the highest binding energy with propylene oxide (15.4 kcal mol^−1^), higher than those of Mn(III) and Ni(II), although not higher than the desorption energy of the products. It is also in accordance with recent studies from the group of North in which they showed that using Cr^III^ salophen co-catalysts allowed the experimental conditions to be lowered to room temperature under specific conditions (use of cardice pellets, increase of the catalyst and co-catalyst mol% up to 2.5 mol% together) (Castro-Osma et al., 2016).

In parallel, the reactivity of the anchored co-catalyst Salophen-tBu-Cr@{NH_2_}-SBA-15 was also investigated at 50, 60, and 80°C (Table 2, entries 1, 2 and 3). For this, the set of parameters, and in particular the substrate:catalyst:co-catalyst molar ratio, was kept identical to that fixed in the study in homogeneous conditions. Furthermore, it is worth noting that Bu_4_NBr was also used as a homogeneous catalyst in that study.

**TABLE 2:** Yield (%) of styrene carbonate obtained with Salophen-tBu-Cr@{NH_2_}-SBA-15

Entry	Co-cat (lewis acid)	cat. (Lewis base)	T (°C)	3 h	7 h	23 h
1	Salophen-tBu-Cr@SBA	Bu_4_NBr	80	58	77	100
2	Salophen-tBu-Cr@SBA	Bu_4_NBr	60	10	32	79
3	Salophen-tBu-Cr@SBA	Bu_4_NBr	50	5	13	47
4^a^	Salophen-tBu-Cr@SBA	Bu_4_NBr	80	n.d.	78	n.d.

Conditions: Styrene oxide (5.6 mmol), Bu_4_NBr (0.1 mmol), supported Salophen-tBu-M (0.05 mmol) in 13 ml of benzonitrile under 11 bars of CO_2_ (initial pressure).

a Reaction conducted in acetonitrile. n.d. = this reaction was not carried out under these specific conditions.

The impact of the heterogenization of the Salophen-tBu-Cr catalyst resulted in a reasonable decrease (from 10 to 20%) in the catalytic activity from the early stage of the reaction. However, in spite of this, the reaction was complete after 23 h at 80°C and the immobilized catalyst was still active even at the lowest temperature (47% styrene oxide conversion after 23 h at 50°C). It should be remembered that such decrease in reactivity between homogeneous and supported catalysts was not observed in the case of the Salophen-tBu-Mn and Salophen-tBu-Ni complexes. On the contrary, reactions conducted in the presence of the Salophen-tBu-Mn@{NH_2_}-SBA-15 and Salophen-tBu-Ni@{NH_2_}-SBA-15 supported catalysts had shown better results compared to those obtained in a homogeneous phase. In the latter examples, the reason proposed for the increased reactivity was related to the non-innocent character of the free amine functions of the support. Indeed, the investigation of the potential co-catalytic activity of the {NH_2_}-SBA-15 support itself, keeping Bu_4_NBr as the main catalyst, had led to a styrene oxide conversion of 86% after 7 h, using the same set of experimental conditions as in the present work. However, the reaction was conducted at 120°C against 80°C in the present study. These results therefore suggest that lowering the reaction temperature from 120°C to 80°C did not allow the amine functions to participate. This is in accordance with the fact that the carbamate obtained by reaction of CO_2_ and amines are more stable at lower temperature (Gouedard et al., 2012). It is therefore expected that the release of CO_2_ will be slowed down at 80°C rather than at 120°C.

**FIGURE 1:**
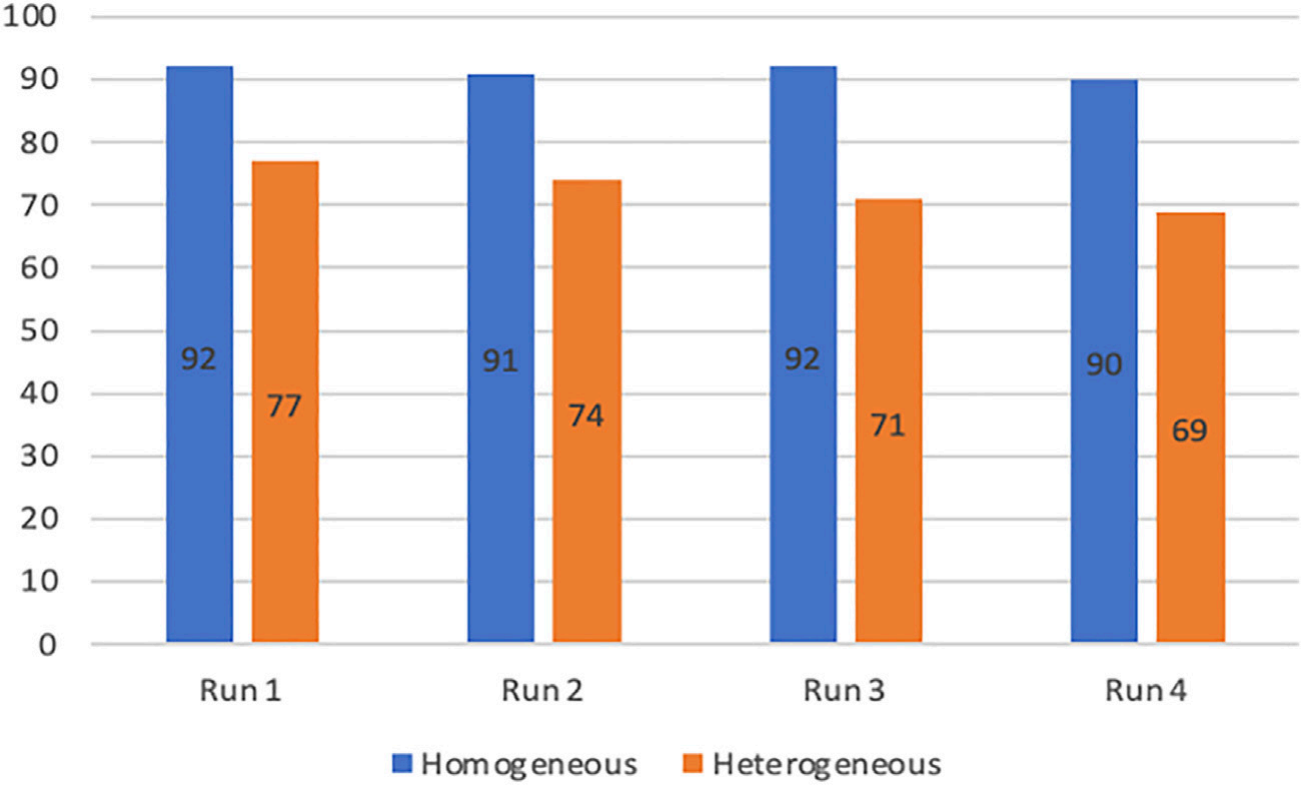
Yields of styrene carbonate obtained after four runs in the presence of Salophen-tBu-Cr (in blue) or Salophen-tBu-Cr@{NH_2_}-SBA-15 (in orange). Conditions: Styrene oxide (5.6 mmol), Bu_4_NBr (0.1 mmol), soluble or supported Salophen-tBu-Cr (0.05 mmol) in 13 ml of benzonitrile under 11 bar of CO_2,_ 7h, 80°C.

In general, the relatively small decrease in reactivity observed in heterogeneous catalytic systems is largely compensated by the ease of catalyst recycling compared to homogeneous systems. The stability of the co-catalyst was thus investigated at 80°C in homogeneous conditions with Salophen-tBu-Cr (4 runs of 7 h with styrene oxide and CO_2_ replenishment without any work-up) and in heterogeneous conditions with Salophen-tBu-Cr@{NH_2_}-SBA-15 (4 runs of 7 h separated by filtration and drying steps). The detailed operating protocols for both experiments are described in the supplementary information section. Yields of styrene carbonate in the corresponding experiments are presented in Figure 1.

The soluble Salophen-tBu-Cr co-catalyst could be successfully re-used without any appreciable loss of performance after respectively four runs, leading to an excellent styrene oxide conversion (from 92 to 90%) for all runs. A weak, but significant decrease, in the conversion (from 77 to 69%) was observed after each run for the supported Salophen-tBu-Cr@{NH_2_}-SBA-15. Regarding the results obtained in homogeneous conditions, this relative loss of reactivity may hardly be explained by a degradation of the catalyst, but was probably the consequence of the recurrent loss of 5 wt% of the supported co-catalyst during the work-up procedure leading to its recovery. Taken together, these results suggest an excellent stability in homogeneous and heterogeneous conditions for Salophen-tBu-Cr, and more generally for this family of complex even in the harsher conditions.

Reactions were also conducted for 7 h at 80°C in acetonitrile in homogeneous (Table 1, entry 10) and heterogeneous conditions (Table 2, entry 4), which is a more desirable solvent than benzonitrile (Byrne et al., 2016). It appears from these experiments, that, taking into account our experimental errors, the yields of styrene carbonate were very close in both solvents (92% with MeCN vs. 91% with PhCN in homogeneous conditions; 78% with MeCN vs. 77% with PhCN in heterogeneous conditions), which means that these solvents have no impact on the reactivity of the catalyst.

## Conclusion

In conclusion, the present work addressed the successful heterogenization of a chromium (III)-salophen complex at the surface of a mesoporous SBA-15 silica as well as its co-catalytic activity in the cycloaddition of CO_2_ onto styrene oxide. The synthesis of the molecular catalyst as well as its strong covalent grafting onto the support were based on simple and efficient experimental protocols. The cycloaddition reaction was investigated in the presence of soluble Bu_4_NBr using the Cr(III) co-catalyst either in solution (homogeneous conditions) or after its immobilization onto the SBA-15 support. In both cases, the presence of the Cr(III)-salophen complex allowed to obtain a dramatic increase of the styrene oxide conversion with respect to the reaction using Bu_4_NBr as the only catalyst, whatever the temperature used. Remarkable yields of styrene carbonate were thus obtained with a nearly complete conversion of styrene oxide after 7 h at 80°C or 23 h at 60°C in solution. This selective catalyst (no other reaction products were detected) also proved to work even at 50°C. Despite a reasonable reactivity decrease after grafting at the surface of the SBA-15, the immobilized catalyst proved to be easily reused after simple filtration and drying steps, without any observable degradation after several runs. From a more general point of view, the use of a Cr^III^ catalyst seems to make it possible to significantly reduce the metal content in this reaction while allowing the process to operate at the lowest temperatures.

## Data Availability

The original contributions presented in the study are included in the article/Supplementary Material, further inquiries can be directed to the corresponding authors.

## Fundings

This study benefited from the support of the project OxCyCat-CO_2_ ANR-17-CE06-0009 of the French Agence Nationale de la Recherche (ANR) for a PhD fellowship to MB, for financial reward for LK and more generally for funding this work.

## Dedication

In memory of our deceased colleague Alain Shum Cheong Sing.
