# Supplementary material for: Chromium-Salophen as a Soluble or Silica-Supported Co-Catalyst for the Fixation of CO2 Onto Styrene Oxide at Low Temperatures
Source: Front Chem. 2021 Oct 29;9:765108. doi: 10.3389/fchem.2021.765108 (PMC8588859; doi:10.3389/fchem.2021.765108)
Supplement: Supplementary file 1 [file DataSheet1.pdf]

Chromium-salophen as a soluble or silica-supported co-catalyst for the fixation of CO_2 onto styrene oxide at low temperatures

Matthieu Balas, Ludivine K/Bidi, Franck Launay*, Richard Villanneau*

Supplementary Material

1 Supplementary Data

1.1 Characterization techniques for the complexes and materials

IR spectra were obtained from KBr pellets on a *Jasco FT/IR-4100* spectrometer with resolution better than 1 cm^{-1}. The ^{1}H NMR solution spectra were recorded in 5 mm o.d. tubes on *Bruker Avance II 300* or *400* spectrometers equipped with a QNP probehead. Chemical shifts were referenced to tetramethylsilane (TMS) for ^{1}H and ^{13}C NMR. Prior to the N_2 sorption analyses at -196°C using an *ASAP-2020 Micromeritics* apparatus, the samples were degassed at 200°C for 6 h. Thermogravimetric analyses (TGA/DSC) were performed under air with a *TA-Instrument SDT Q600*, between 20 and 700°C (air flow rate: 100.0 $mL.min^{-1}$, slope: 10°$C.min^{-1}$). High-resolution mass spectra (HRMS) were measured by the Service de Spectrométrie de Masse of Sorbonne Université. Gas chromatography analyses were performed on a Shimadzu NEXIS-GC-2030 AF instrument equipped with a split/splitless (SPL) injector, a flame ionization detector and a GC Capillary Column SH-Rxi-5MS (ID: 0.25 mm; film thickness: 0.25 µm; Length: 30 m) using He as a vector gas (1.5 mL min^{-1}) and the following temperature conditions: ramp from 70 to 250°C at 10°C min^{-1}, then an isotherm at 250°C during 10 min). The X-ray diffraction (XRD) patterns were recorded on a Bruker AXS D8 diffractometer operating at 30 kV and 30 mA and using a Cu Kα radiation (α = 1.54184 Å) as X-ray source. For the low angles diffraction experiments, the data were collected in the 2θ range from 0.5 to 5° with a step of 0.02° and a counting time of 6 s/step.

1.2. Protocols for the catalysts synthesis

Synthesis of N,N'-bis(3,5-di-tert-butylsalicylidene)-1-carboxy-3,4-phenylene-diamine (H_2Salophen)

3,4-Diaminobenzoic acid (1 g, 1 eq, 6.6 mmol) was dissolved in 20 mL of dry tetrahydrofuran (THF) under argon in a two-neck round-bottom flask connected to a condenser. In a second flask, 3,5-di-*tert*-butyl-2-hydroxobenzaldehyde (3.1 g, 2 eq, 13.1 mmol) was dissolved in 20 mL of dry THF under argon. The second solution was transferred to the first dropwise under vigorous stirring. A solution of 0.7 M $ZnCl_2$ in dry THF was then added dropwise and the resulting mixture was stirred under reflux for 15 min. After cooling at room temperature and evaporation of the THF, the residue was washed by 20 mL of methanol. The yellow solid was then collected and rinsed 2 times with 20 mL methanol. (H_2Salophen) (yield: 2.97g, 80%). HRMS [**H_2Salophen**+H]$^+$ (ESI, Fig. S1) : m/z = 585.37.

Synthesis of N,N'-bis(3,5-di-tert-butylsalicylidene)-1-carboxy-3,4-phenylene-diamine-chromium(III)chloride (Salophen-tBu-CrCl)

H_2Salophen ligand (0.290 g, 1 eq, 0.50 mmol) was dissolved in 25 mL of dry THF under argon in a two-neck round-bottom flask connected to a condenser. Then, a solution of [$CrCl_3(THF)_3$] (0.190 g, 1 eq, 0.50 mmol) in 25 mL of dry THF was prepared and transferred into a dropping funnel under argon. This solution was added dropwise and the resulting mixture was stirred under reflux for 15 min. After cooling at room temperature, the solution was evaporated under vacuum leading to a dark red solid (Salophen-tBu-Cr) (yield: 0.179 g, 53%).

Preparation of {NH_2}-SBA-15.

SBA-15 was pre-formed via a classical sol-gel procedure described elsewhere.[i] SBA-15 was then functionalized with 3-aminopropyltriethoxysilane as described previously, targeting 3 mmol.g^{-1}).[i] The obtained support was then characterized by TGA, XRD and N_2 physisorption. The thermogravimetric curve of {NH_2}-SBA-15 performed under air from room temperature up to 700°C shows two weight losses. The first one (*ca.* 3%), below 100°C, can be attributed to the loss of water molecules weakly adsorbed on the silica surface whereas the second one (13%, 100-700°C) was assigned to the loss of aminopropyl functions. This TGA analysis showed that {NH_2}-SBA-15 would be functionalized with 2.3 mmol of {NH_2}.g^{-1} (*c.a.* 77 % incorporation yield).

Covalent grafting of Salophen-tBu-Cr onto {NH_2}-SBA-15.

{NH_2}-SBA-15 (1 g, [-RNH_2] = 2.3 mmol g^{-1}) and Salophen-tBu-Cr (0.200 g, 0.30 mmol) were dried under vacuum overnight in two different schlenk tubes. Then, Salophen-tBu-Cr was dissolved in dry dichloromethane (DCM, 7 mL). In parallel, 1-hydroxy-1H-benzotriazole (HOBT, 0.022 g, 0.15 mmol) and *N,N'*-dicyclohexylcarbodiimide (DCC, 0.143 g, 0.68 mmol) were added to the solution of Salophen-tBu-Cr. The mixture was stirred for 40 min at room temperature. Meanwhile, 8 mL of DCM were added to {NH_2}-SBA-15. After 40 min, the previous mixture of Salophen-tBu-Cr, HOBT and DCC was transferred to the suspension of {NH_2}-SBA-15 and the mixture refluxed for 6 h under N_2. Then, the solvent was evaporated until dryness and the resulting orange powder was washed successively with refluxing methanol and acetone using a Soxhlet, respectively for 3 days and 24 h. Salophen-tBu-Cr@{NH_2}-SBA-15 (1.1 g) was thus obtained as a bright orange powder (Cr $\%_{weight}$ = 0.8 % (5.6% mol Cr/mol –NH_2) corresponding to a Cr grafting yield of 41 %).

1.3. Protocols for the catalysis tests

After each catalytic test, the resulting solutions or suspensions were analysed by gas chromatography (GC, see details in part 1 of this section) after dilution (20 μL of the reaction mixture diluted in 10 mL of CH_2Cl_2).

Experiments in homogeneous conditions (including stability test)

In a 50 mL Teflon container, 0.7 mL of styrene oxide (6.1 mmol), 0.031 g (0.100 mmol) of $^{n-}Bu_4NBr$ and 1 mL of *p*-xylene (internal standard) were dissolved in 13.3 mL of benzonitrile. Except for the tests performed in the absence of co-catalyst, 0.032 g of Salophen-CrCl (0.05 mmol)) was added and the resulting mixture was stirred for 5 min at room temperature. Then, the autoclave was pressurized

at 11 bar of CO_2 (corresponding to 41 mmol). The temperature was then increased up to 50-80°C in 20 min. Heating was then prolonged for 3, 7 or 23 h and the reaction was quenched by cooling the autoclave into a water-ice mixture.

For the stability test, the reaction was carried out for 7 h, then quenched as described above. After analysis by GC, a new batch of styrene oxide (0.7 mL, 6.1 mmol) was introduced in the recovered Teflon container (that still contained the $^{n-}Bu_4NBr$ catalyst and the co-catalyst) and the reaction mixture was stirred for 5 min at room temperature. Then, the autoclave was pressurized with CO_2 as described before. This procedure was repeated 3 times.

Experiments with the supported complexes (including recyclability test)

The protocols were identical with the exception of the mass of co-catalysts added. 0.325 g of Salophen-Cr@{NH_2}-SBA-15, corresponding to 0.05 mmol of immobilized metal salophen complexes, was suspended in the benzonitrile solution. Then, the autoclave was pressurized as described before. After 3, 7 or 23 h, the reaction was quenched by cooling the autoclave into a water-ice mixture, and the reaction mixture was filtered on a büchner funnel, in order to separate the supported complex.

For the recyclability test, the reaction was carried out for 7 h and the solid recovered after each attempt was carefully washed by acetone, dried in an oven at 50°C for 12 h and weighted to check the mass after each run. The solid was then added in a Teflon vessel containing 0.7 mL of styrene oxide (6.1 mmol), 0.031 g (0.100 mmol) of $^{n-}Bu_4NBr$ and 1 mL of *p*-xylene (internal standard) dissolved in 13.3 mL of benzonitrile. This procedure was repeated three times.

2 Supplementary Figures

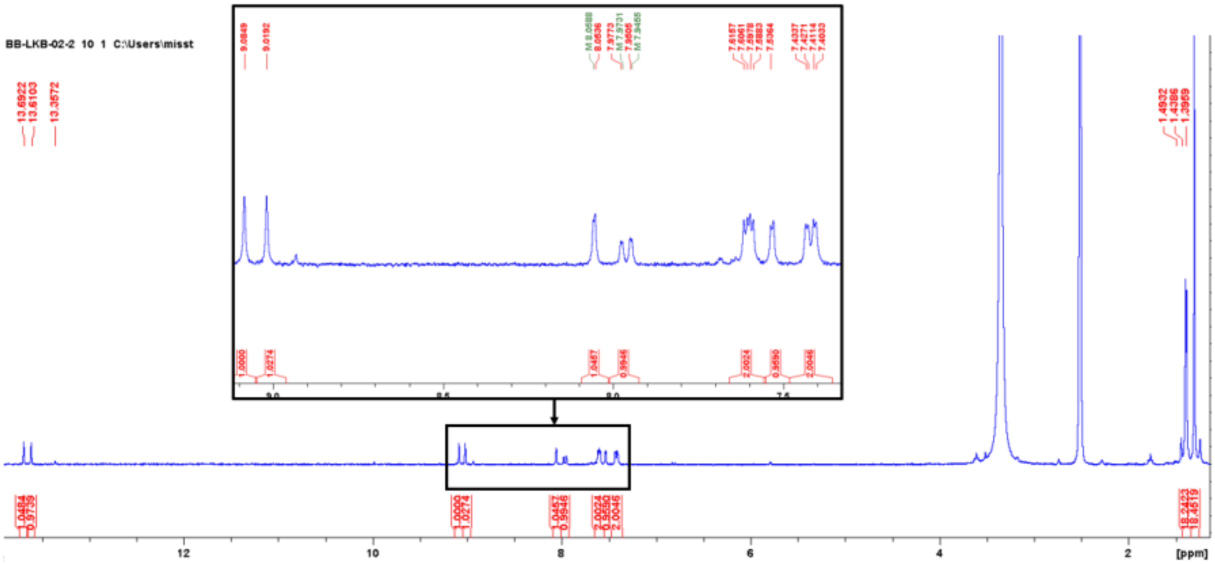

Figure S1: ^{1}H NMR spectrum of H_2Salophen. NMR ^{1}H (DMSO - d6): δ13.69 (1H, s, C-OH), 13.61 (1H, s, C-OH), 9.08 (1H, s, H-C=N), 9.01 (1H, s, H-C=N), 8.05 (1H, s, C-H), 7.97 (1H, d, C-H), 7.95 (1H, d, C-H), 7.61 - 7.58 (2H, m, C-H), 7.53 (1H, d, C-H), 7.43 - 7.4 (2H, m, C-H), 1.39.

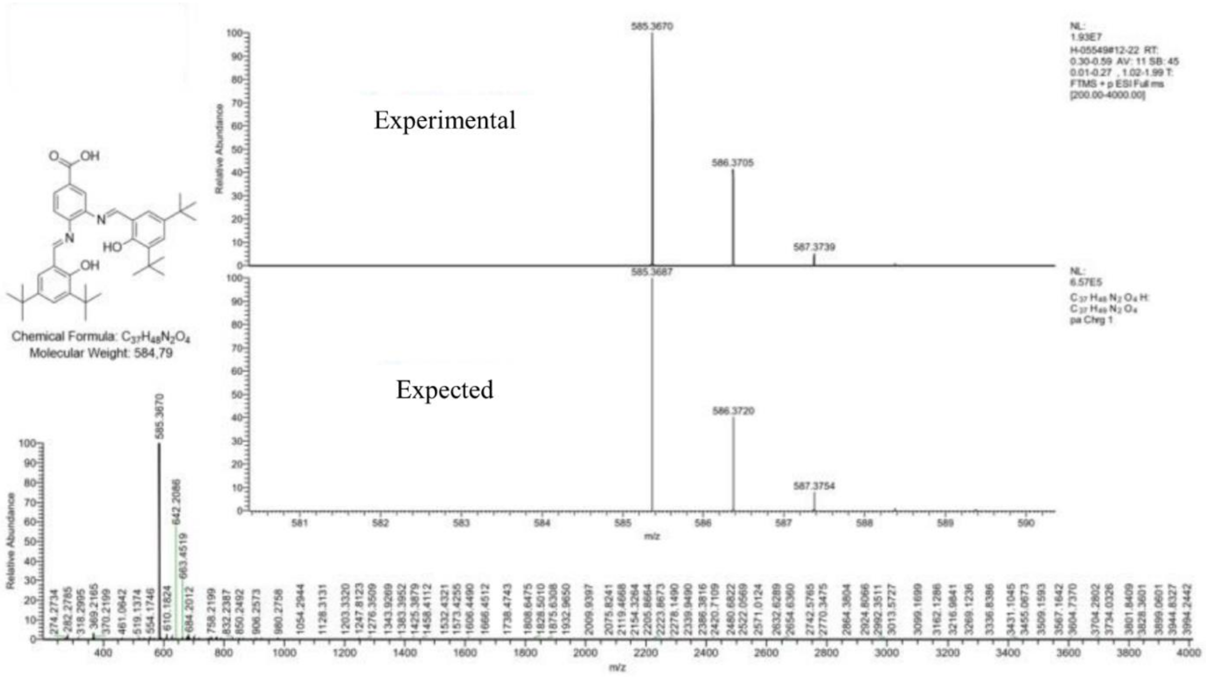

Figure S2: HRMS (ESI) spectrum for H_2Salophen ligand calculated for $C_{37}H_{48}N_2O_4$ (m/z ($[M+H]^+$) = 585.37.

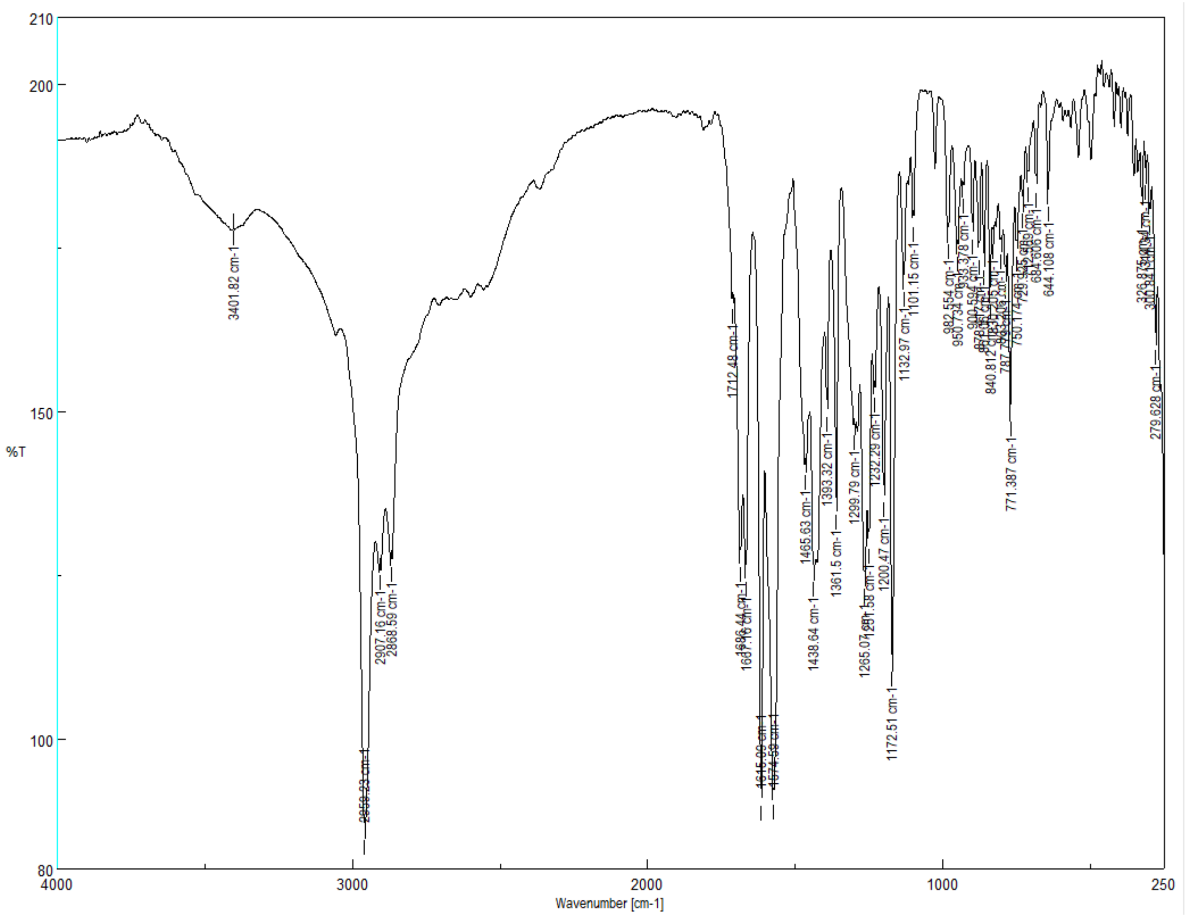

Figure S3: Infrared spectrum for H_2salophen ligand (KBr pellets). IR (cm^{-1}): 3425 (w), 2958 (s), 2907 (m), 2869 (m), 1712 (w), 1686 (m), 1667 (m), 1615 (s), 1574 (s), 1465 (m), 1439 (m), 1361 (m), 1300 (m), 1265 (m), 1252 (m), 1200 (m), 1172 (s), 1132 (w), 1026 (w), 982 (w), 951 (w), 900 (w), 840 (w), 788 (w), 771 (m), 644 (w) cm^{-1}.

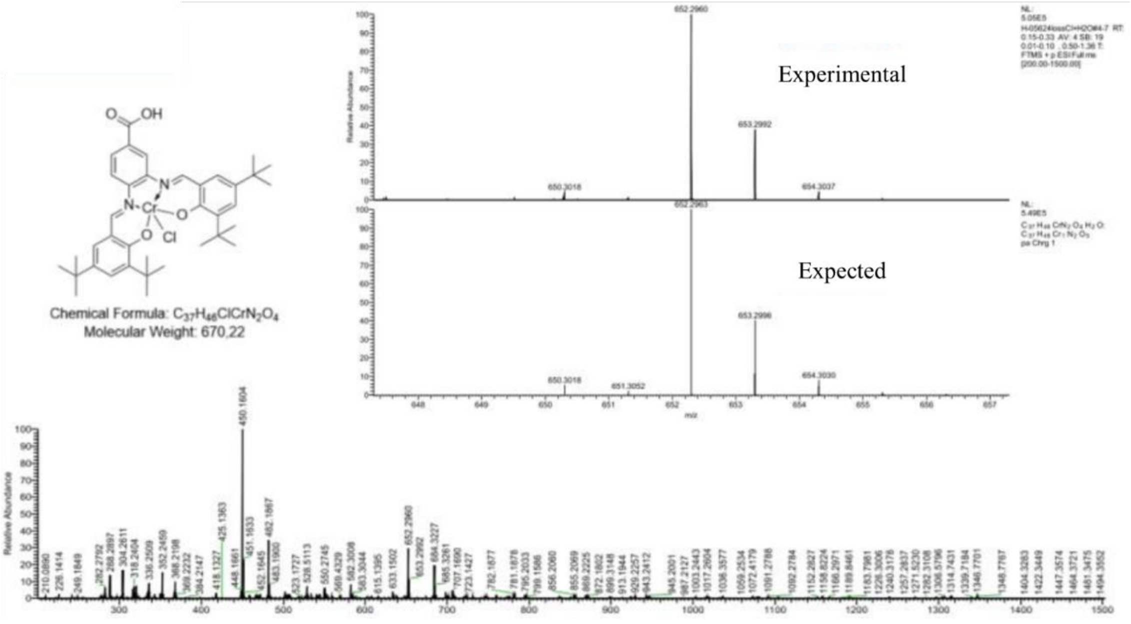

Figure S4: HRMS (ESI) spectrum for Salophen-tBu-CrCl calculated for $C_{37}H_{48}N_2O_5Cr$ (m/z ($[M-Cl+H_2Ol]^+$) = 652.30.

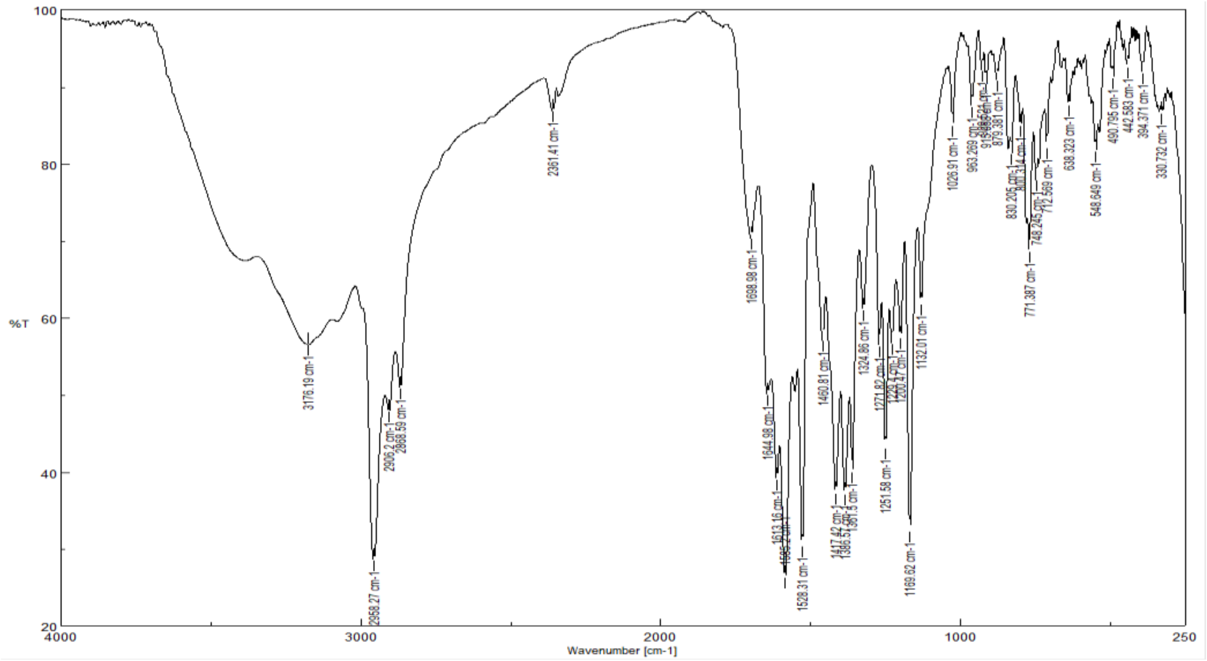

Figure S5: Infrared spectrum for Salophen-tBu-CrCl (KBr pellets). IR (cm^{-1}): 3716 (s), 2958 (s), 2906 (m), 2869 (m), 1699 (m), 1645 (m), 1615 (m), 1585 (s), 1528 (s), 1461 (m), 1417 (m), 1386 (m), 1361 (m), 1324 (w), 1271 (m), 1252 (m), 1200 (m), 1170 (s), 1132 (w), 1027 (w), 963 (w), 915 (w), 879 (w), 830 (w), 771 (m), 748 (w), 638 (w), 549 (w) cm^{-1}.

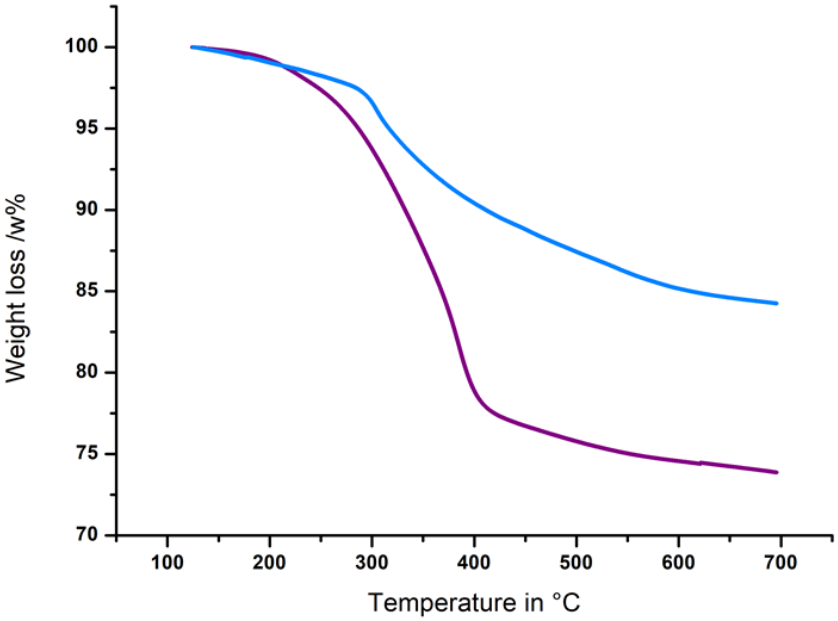

Figure S6: Weight loss % (thermogravimetric analysis, TGA) for $\{NH_2\}$-SBA-15 (blue) and Salophen-tBu-Cr@$\{NH_2\}$-SBA-15 (purple). All curves were normalized at 100°C.

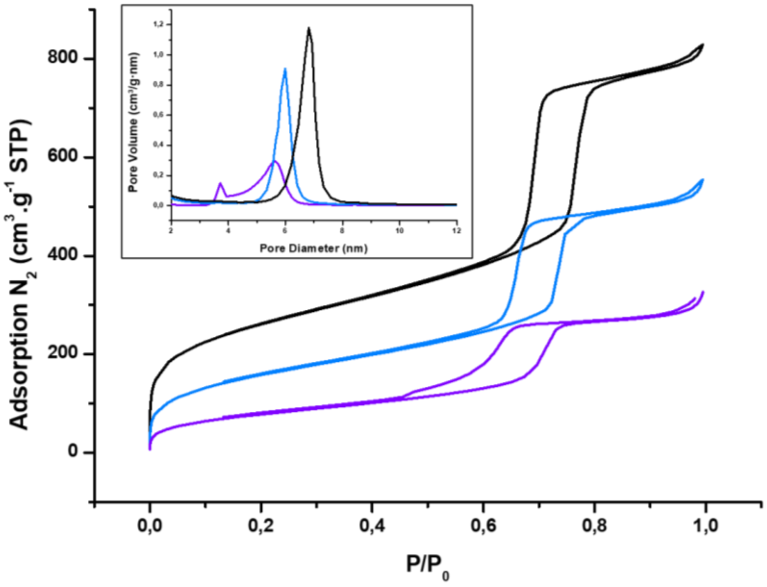

Figure S7: Nitrogen adsorption-desorption isotherms (77K) of SBA-15 (black), {NH_2}-SBA-15 (blue) and Salophen-tBu-Cr@{NH_2}-SBA-15 (purple) with pore size distribution curves (inset).

Table S1: *Textural parameters for SBA-15 and its functionalized derivatives*

Sample	*S_{BET} ($m^2.g^{-1}$)*	*Pore Vol. ($cm^3.g^{-1}$)*	*Average Pore diameter (nm)*
SBA-15	*900*	*1.12*	*6.2*
{NH_2}-SBA-15	*530*	*0.69*	*5.7*
{Salophen-tBu-Cr}-SBA-15	*310*	*0.44*	*-[a]*

[a] The pore sizes distribution curve is characterized by two maximum values (see Figure S7). It is not meaningful to give an average pore diameter value in that case.

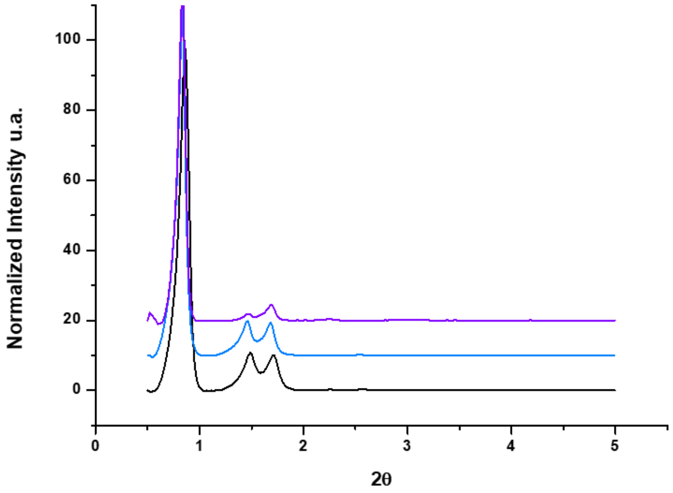

Figure S8: X-ray diffraction patterns for SBA-15 (black), {NH_2}-SBA-15 (blue) and Salophen-tBu-Cr@{NH_2}-SBA-15(purple), showing the high hexagonal structuration of the porosity of all materials (presence of the (100), (110) and (200) reticular planes characteristic of the SBA-15 structure).

---

[i] R. Villanneau, A. Marzouk, Y. Wang, A. Ben Djamaa, G. Laugel, A. Proust, F. Launay, *Inorg. Chem.* **2013**, *52*, 2958-2965.
